# External environment sensitive circularly polarized luminescence properties of a chiral boron difluoride complex†

**DOI:** 10.1039/d2ra07386b

**Published:** 2022-12-06

**Authors:** Masahiro Ikeshita, Hongxi He, Maho Kitahara, Yoshitane Imai, Takashi Tsuno

**Affiliations:** Department of Applied Molecular Chemistry, College of Industrial Technology, Nihon University Narashino Chiba 275-8575 Japan ikeshita.masahiro@nihon-u.ac.jp tsuno.takashi@nihon-u.ac.jp; Department of Applied Chemistry, Faculty of Science and Engineering, Kindai University 3-4-1 Kowakae Higashi-Osaka Osaka 577-8502 Japan y-imai@apch.kindai.ac.jp

## Abstract

A chiral Schiff-base boron difluoride complex bearing a diethylamino group was synthesized. Its photophysical properties were investigated and compared with those of its non-substituted analogue. The complex was found to exhibit solvatofluorochromism with bluish-white emission in moderately polar solvents and intense blue emission in nonpolar solvent. Circularly polarized luminescence (CPL) properties were also examined and it was found that the absolute value of the luminescence dissymmetry factor (*g*_lum_) increases significantly in the KBr-dispersed pellet state compared to the solution state. Notably, CPL intensity of the complex enhanced approximately three times upon addition of CH_3_SO_3_H in CH_2_Cl_2_. Density functional theory (DFT) calculations were conducted to further understand the photophysical properties.

## Abstract 2

Circularly polarized luminescence properties of chiral boron difluoride complexes bearing a diethylamino group successfully controlled by external environment.

## Graphical abstract

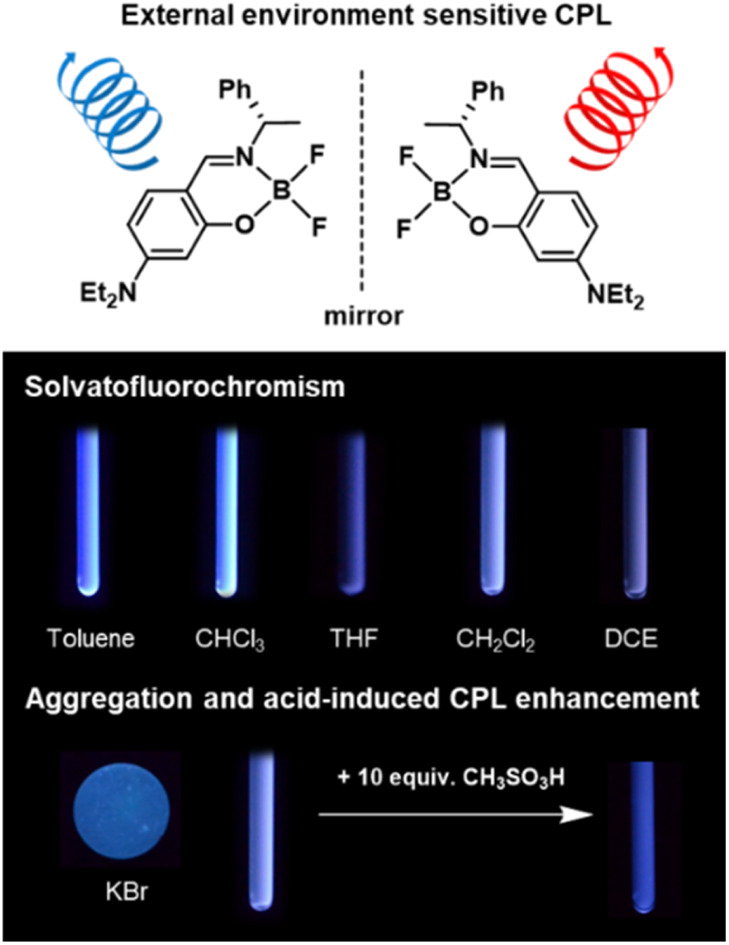

## Introductions

Circularly polarized luminescence (CPL)^1–4^ which is defined as differential emission of left- *versus* right-circularly polarized light have attracted increasing attentions over the past decade as an important phenomenon with potential applications in 3D optical displays,^5^ biological probes,^6^ asymmetric synthesis,^7^ as well as CPL lasers.^8^ Among the various CPL-active materials, small organic molecules (SOMs)^9^ have drawn growing interest owing to the potential application for circularly polarized organic light-emitting diodes (CP-OLEDs).^10^ Numerous examples of CPL-SOMs with chiral frameworks such as helicenes,^11^ cyclophanes^12^ and binaphthyls^13^ have been developed and several studies have been reported to establish guidelines for designing molecules that exhibit high CPL efficiency.^11*d*–*g*,12*g*,13*f*^ Controlling CPL characteristics of CPL-SOMs is an important subject in the development of advanced information technologies.^14^ One of the strategy to achieve CPL control of SOMs is to design molecules that are sensitive to conformational changes in response to the external environment. To date, a variety of SOMs have been developed in which CPL properties can be controlled depending on the external environment, such as solvent,^15^ dispersed matrix^16^ and pH.^17^

Organoboron complexes have received increasing attention in recent years due to their efficient and tunable luminescent properties.^18^ Such characteristics have led to their wide utilization in optical and optoelectronic devices, including organic light-emitting diodes (OLEDs).^19^ Boron difluoride complexes, one of the families of organoboron complexes, have been recognized as promising materials for CPL-SOMs due to their ease of preparation and modification.^20^ Various CPL-active boron difluoride complexes^21^ containing conjugated π-systems including axial chirality,^21*a*,*c*,*i*^ helical chirality^21*b*,*d*,*h*^ and planar chirality^21*e*–*g*^ have been reported.

**Fig. 1:**
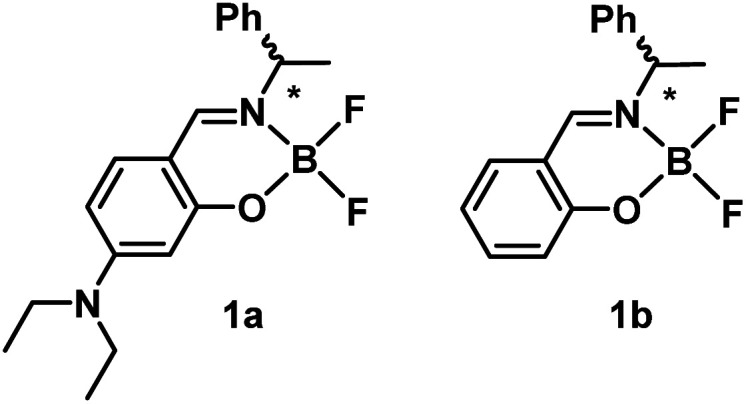
Structures of the boron difluoride complexes studied in this work.

As part of our program aimed at the creation of novel functional materials with CPL-activities, we have developed organic and organometallic complexes bearing chiral Schiff-base ligands.^16*e*,21*j*,22^ Previously, we reported that boron difluoride complexes with chiral Schiff-base ligands exhibit multi-colour CPL properties in dilute solution and in the drop cast film state.^21*j*^ In the present work, we aimed to develop novel boron difluoride complexes with controllable CPL properties depending on the external environment for further applications. To this purpose, complex 1a bearing a diethylamino group was newly designed and the photophysical properties of the complex were compared with the non-substituted analogue 1b (Fig. 1). As a result, it was found that intensity and colour of CPL can be controlled by varying solvent polarity, dispersed matrix and pH of the solution. Theoretical calculations revealed a relationship between their structures and the photophysical properties including CPL-activities. Herein we describe the synthesis, structure and photophysical properties of chiral Schiff-base boron difluoride complexes with a focus on its tunable CPL properties.

## Results and discussion

### Synthesis and structures

**Scheme 1:**
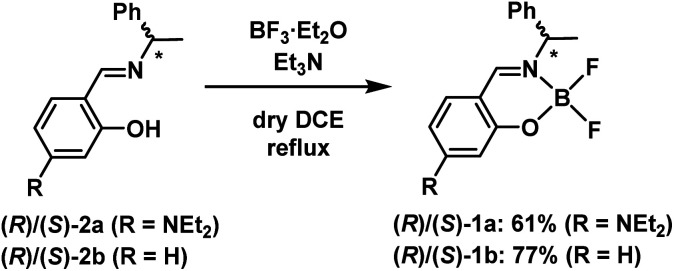
Synthesis of chiral boron difluoride complexes (*R*)/(*S*)-1a and 1b.

The chiral boron difluoride complexes (*R*)-1a and (*S*)-1a were successfully synthesized by the reaction of BF_3_·OEt_2_ with optically pure Schiff-base ligands (*R*)-2a and (*S*)-2a bearing a diethylamino group in dry 1,2-dichloroethane (DCE) according to the reported procedure (Scheme 1).^20*d*^ The non-substituted analogues (*R*)-1b and (*S*)-1b were also prepared as reference compounds from the corresponding optically pure Schiff-base ligands (*R*)-2b and (*S*)-2b by the same synthetic methods. The newly synthesized compounds 1a and 2a were successfully characterized by ^1^H and ^13^C nuclear magnetic resonance (NMR) spectroscopy (Fig. S1 and S3, ESI†), infra-red (IR) spectroscopy, high-resolution mass spectrometry (HRMS) and elemental analysis, respectively.

**Fig. 2:**
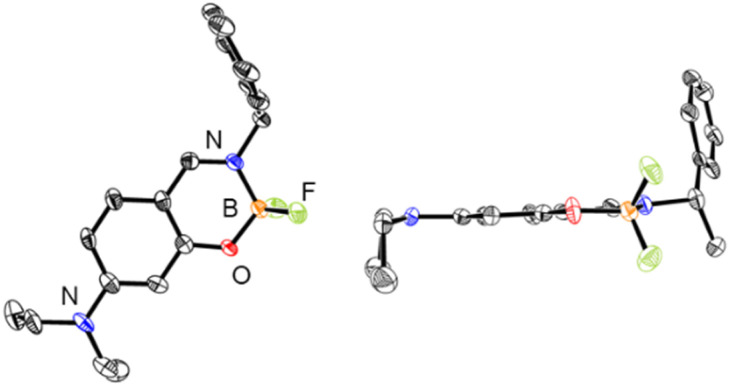
ORTEP representations of (*S*)-1a. Left figures: overhead views. Right figures: side views.

Single crystals of (*S*)-1a were obtained by recrystallization from a CH_2_Cl_2_/EtOH solution and the molecular structure was unequivocally established by X-ray diffraction (XRD) analysis at 113 K. The details of the crystal data and the structure refinement are presented in Table S1 (ESI†). ORTEP^23^ drawings of (*S*)-1a are presented in Fig. 2. The boron atoms in (*S*)-1a adopt a typical tetrahedral geometry to form a six-membered ring which is similar to the previously reported boron difluoride complexes.^20^ The packing structure and major interactions in the lattice are shown in Fig. S5 (ESI†). (*S*)-1a crystallizes in the chiral monoclinic space group *P*2_1_. In the lattice, complex (*S*)-1a was fixed three-dimensional intermolecular H–F bonds.

### Photophysical properties

**Fig. 3:**
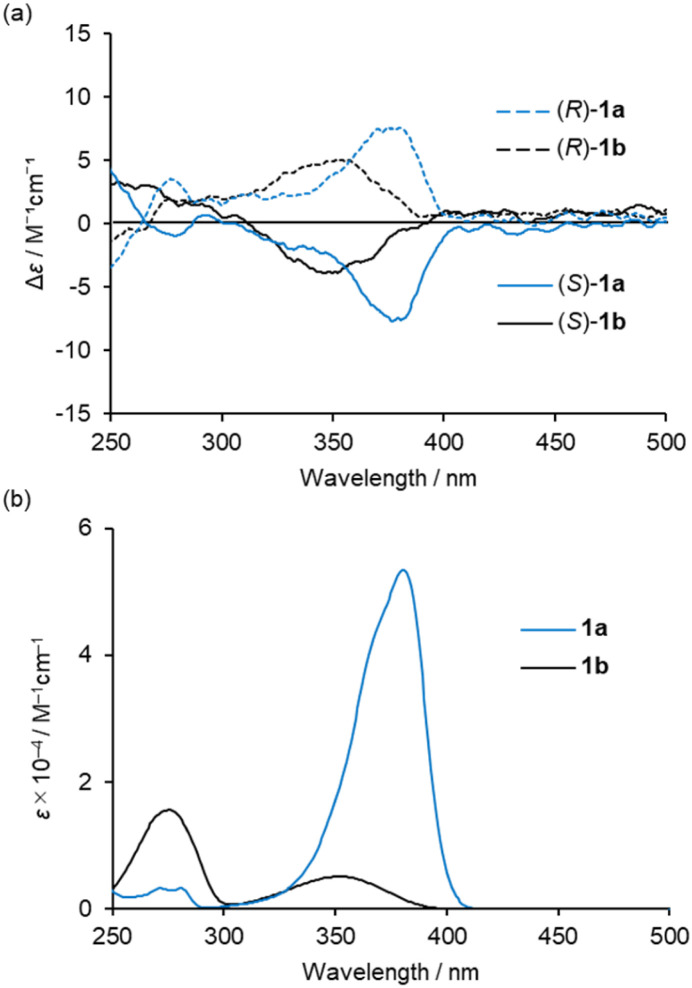
(a) CD and (b) UV-vis spectra of 2.0 × 10^−4^ M solutions of 1a and 1b in CH_2_Cl_2_ at 298 K.

Circular dichroism (CD) and UV-vis absorption spectra of (*R*)-1a and (*S*)-1a were recorded in CH_2_Cl_2_ solution at room temperature (Fig. 3a and b). The spectra of complexes (*R*)-1b and (*S*)-1b under the same conditions are also shown for comparison.^20*d*^ (*R*)-1a and (*S*)-1a showed mirror image CD spectra with their maxima matching the maxima of the UV-vis absorption spectra. The low energy band of 1a was increased and bathochromically shifted compared to that of 1b in both CD and UV-vis absorption spectra. This is attributed to the participation of n–π* transition character from the lone pair of the nitrogen atom of the diethylamino group. The |*g*_abs_| (=Δ*ε*/*ε*) values around the absorption maxima in the low energy region are calculated to be 1.4 × 10^−4^ (380 nm) for 1a and 1.0 × 10^−3^ (348 nm) for 1b, respectively. These results indicate that the participation of n–π* increases *ε* value, resulting in a decrease in the |*g*_abs_| value. Further consideration will be discussed in a later section with the results of the theoretical calculations.

**Fig. 4:**
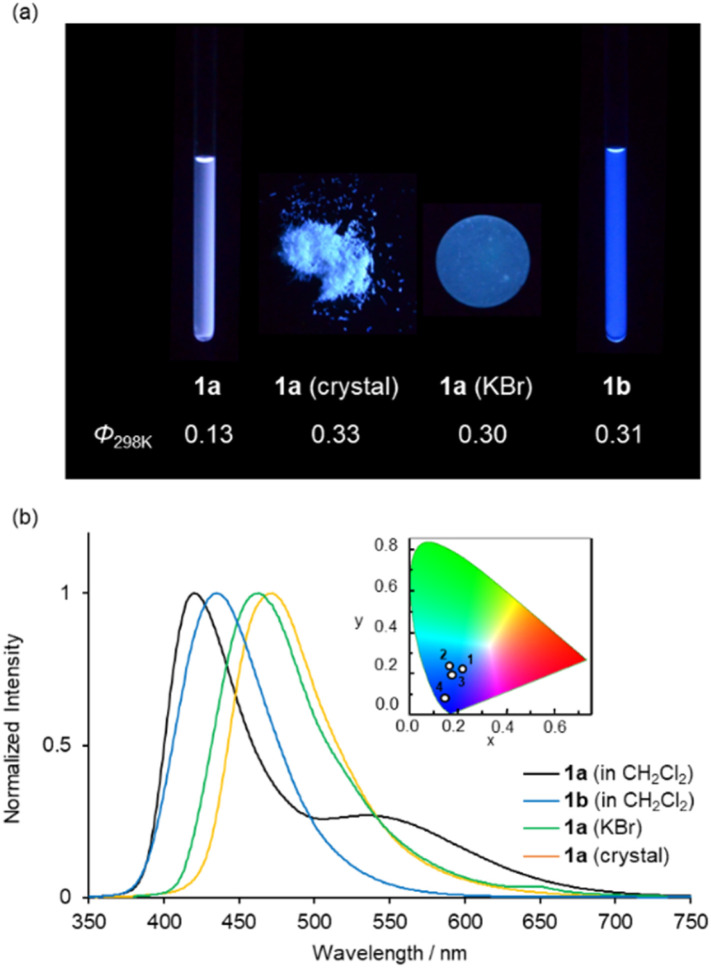
(a) Photographs under UV illumination at 365 nm and (b) normalized emission spectra of 1a and 1b in CH_2_Cl_2_ (2.0 × 10^−4^ M), crystalline and in the KBr-dispersed pellet state at 298 K (*λ*_ex_ = 350 nm). The insets in (b) show the CIE colour coordinates of the emissions (1, 1a in CH_2_Cl_2_; 2, 1b in CH_2_Cl_2_; 3, 1a (KBr); 4, 1a (crystal)).

**Scheme 2:**
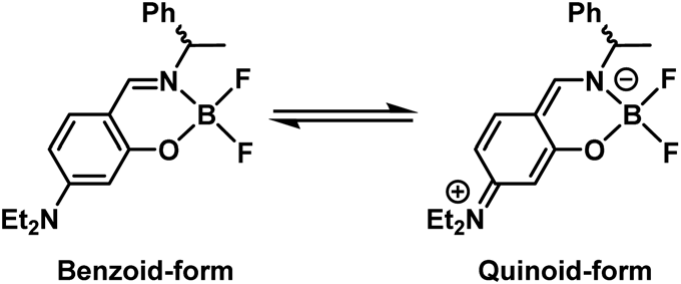
Plausible equilibrium of the resonance structures of complex 1a in the excited state.

Complex 1a exhibited blue to bluish-white luminescence with moderate emission quantum efficiencies (*Φ*) under UV excitation at room temperature in dilute CH_2_Cl_2_ solution, in the crystalline and in the KBr-dispersed pellet state, respectively (Fig. 4a). The photophysical data for complexes 1a and 1b are presented in Table 1. The emission spectra in each state are shown in Fig. 4b. In the solution state, complex 1a exhibited two emission bands around 420 and 540 nm, while 1b showed only one emission band around 430 nm under the same condition. The emission band around 540 nm in complex 1a is attributed to the *para*-quinoidal resonance structure in the excited state (Scheme 2).^24^ The first emission band in 1a showed a clear bathochromic shift in the solid state (*λ*_max_ = 472 nm for crystal and *λ*_max_ = 463 nm for crystal) compared to that of in solution state (*λ*_max_ = 420 nm). The CIE colour coordinates plotted on the CIE1931 chromaticity chart^25^ (Fig. 4b, inset) indicate that the emission colour of 1a and 1b varies between the blue to white region in each state.

**Table 1:** Photophysical data for complexes 1a and 1b^a^

Compound	Medium	*λ*_abs_ [nm]	*λ*_max_ b [nm]	*Φ* b ^,^ c	|*g*_lum_|^d^	CIE (*x*, *y*)^b^
1a	CH_2_Cl_2_	281, 381	420, 539	0.13	2.8 × 10^−4^ (426 nm)	0.23, 0.21
	CH_2_Cl_2_ + CH_3_SO_3_H (10 eq.)	267, 346	423	0.12	9.0 × 10^−4^ (426 nm)	0.17, 0.09
	Toluene	279, 379	414	0.59	2.3 × 10^−4^ (423 nm)	0.17, 0.07
	CHCl_3_	282, 381	417, 534	0.49	2.6 × 10^−4^ (418 nm)	0.18, 0.12
	DCE	281, 380	421, 546	0.07	2.0 × 10^−4^ (423 nm)	0.25, 0.22
	THF	279, 375	417, 550	0.04	2.4 × 10^−4^ (424 nm)	0.22, 0.19
	Crystal	—	472	0.33	—	0.16, 0.23
	KBr pellet	—	463	0.30	2.4 × 10^−3^ (461 nm)	0.17, 0.20
1b	CH_2_Cl_2_	354^e^	433^e^	0.32^e^	1.0 × 10^−3^ (433 nm)	0.15, 0.08

a Data were obtained from a 2.0 × 10^−4^ M solution, crystals or KBr-dispersed pellets at 298 K.

b *λ*_ex_ = 350 nm.

c Luminescent quantum efficiencies measured using the absolute method with an integrating sphere.

d The |*g*_lum_| values around emission peak maxima are listed.

e The data for 1b (reported in ref. 20*d*) are provided for comparison.

**Fig. 5:**
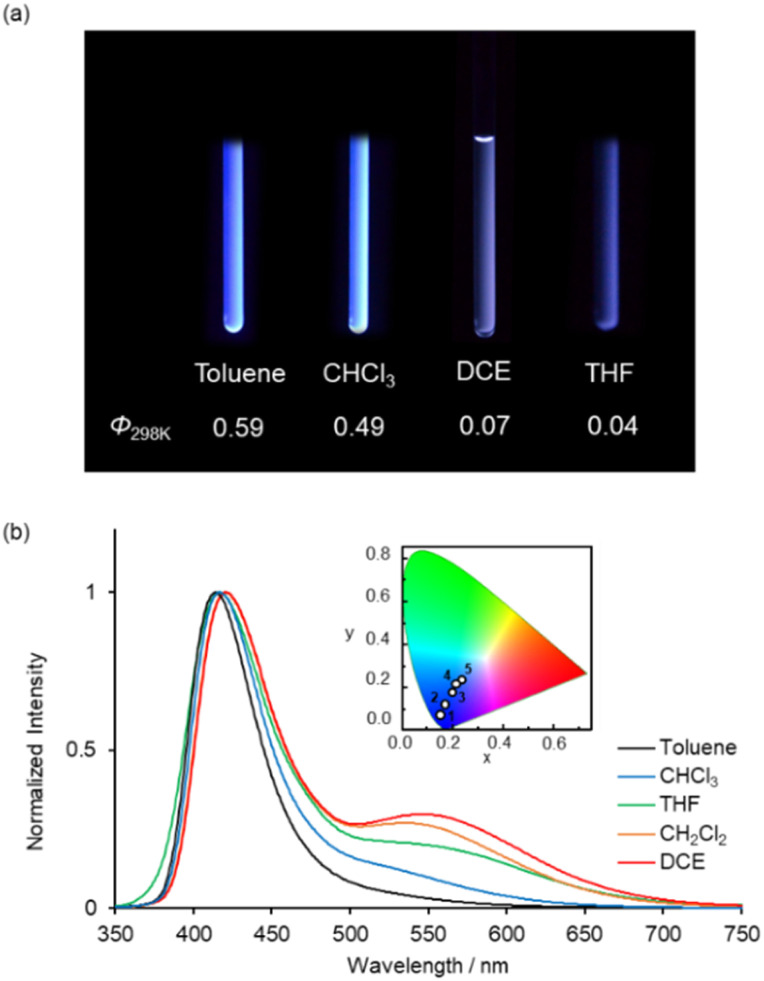
(a) Photographs under UV illumination at 365 nm and (b) normalized emission spectra of 2.0 × 10^−4^ M solutions of (*S*)-1a in various organic solvents at 298 K (*λ*_ex_ = 350 nm). The insets in (b) show the CIE colour coordinates of the emissions (solvents: 1, toluene; 2, CHCl_3_; 3, THF; 4, CH_2_Cl_2_; 5, DCE).

While it has been reported that 1b exhibits identical fluorescent properties in any solvent,^20*d*^1a exhibits solvatofluorochromism depending on the polarity of the solvents. Fig. 5a shows photographs of 1a in various organic solvents under UV irradiation. Bluish-white emissions were observed in moderately polar solvents such as CHCl_3_, DCE and THF as well as CH_2_Cl_2_. In nonpolar solvents like toluene, 1a showed intense blue emission with a high *Φ* value (0.59) (Fig. 5a). In highly polar solvents (CH_3_CN, acetone, DMF and MeOH), 1a exhibited weak blue emission with a low *Φ* value (0.01) (Fig. S6 and Table S2, ESI†). The UV-vis absorption spectra of 1a in various organic solvents are shown in Fig. S7 (ESI†), where identical absorption spectra were observed in all solutions. From these results, we conclude that the polarity of the solvents affects the stability of the resonance structures (benzoid- and quinoid-form) in the excited state which is the key to the dual emission properties of 1a.

**Fig. 6:**
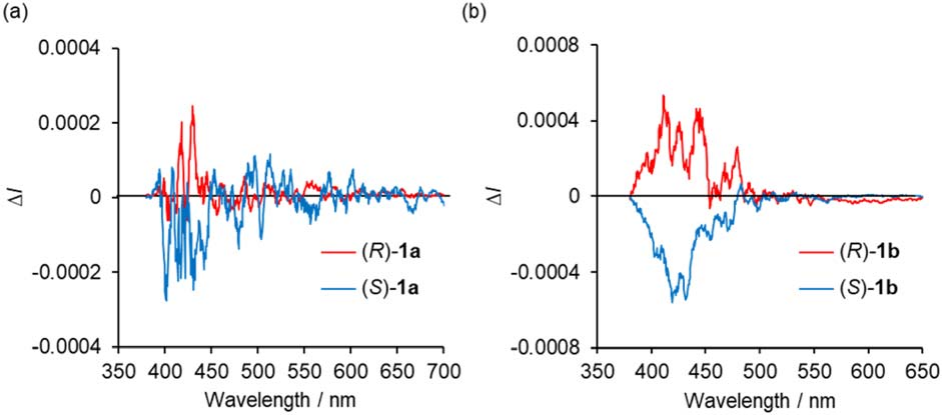
CPL spectra of (*R*)-and (*S*)- (a) 1a and (b) 1b in CH_2_Cl_2_ (2.0 × 10^−4^ M) at 298 K (*λ*_ex_ = 350 nm).

**Fig. 7:**
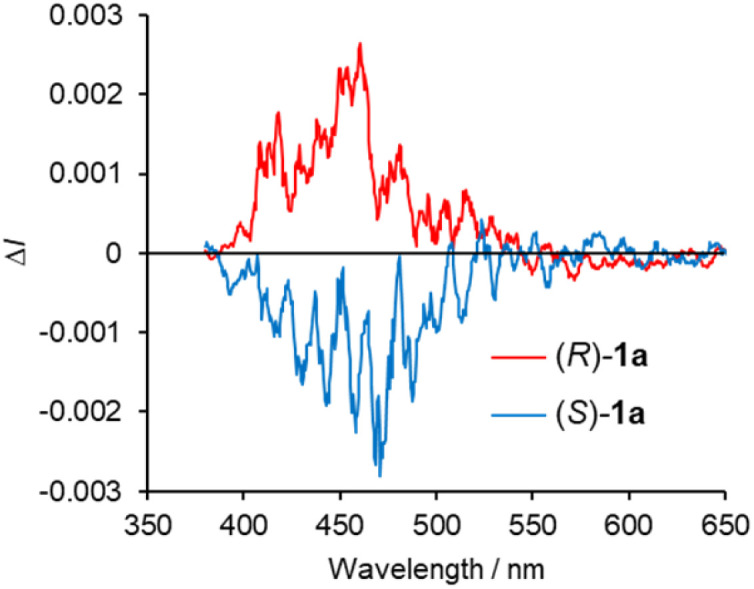
CPL spectra of (*R*)-and (*S*)-1a in the KBr-dispersed pellet state at 298 K (*λ*_ex_ = 350 nm).

The CPL spectra of the enantiomeric samples 1a and 1b in dilute organic solutions show mirror image spectra (Fig. 6 and S8, ESI†), and the emission peak maxima of the CPL signals correspond well to the emission spectra taken under the same measurement conditions (Fig. 4b and 5b). In general, the efficiency of CPL is usually quantified by means of the luminescence dissymmetry factor (*g*_lum_ = 2Δ*I*/*I* = 2(*I*_L_ − *I*_R_)/(*I*_L_ + *I*_R_), in which *I*_L_ and *I*_R_ are the intensity of left- and right-circularly polarized luminescence).^26^ The |*g*_lum_| values around the maximum emission wavelength in CH_2_Cl_2_ solution are calculated to be 2.8 × 10^−4^ (426 nm) for 1a and 1.0 × 10^−3^ (433 nm) for 1b, respectively, which are typical values for small organic and organometallic molecules.^27^ The CPL spectra of (*R*)-1a and (*S*)-1a in toluene, CHCl_3_, DCE and THF also showed *g*_lum_ values of the 10^−4^ order (Table 1 and Fig. S8, ESI†). The decrease in the |*g*_lum_| value caused by the introduction of a diethylamino group corresponds to the decrease in the |*g*_abs_| value calculated from the CD spectra, which will be also discussed with the results of theoretical calculations described below. The CPL spectra of (*R*)-1a and (*S*)-1a were also recorded in the KBr-dispersed pellet state, showing clear mirror image signals. Their maximum emission |*g*_lum_| values are calculated to be 2.4 × 10^−3^ (461 nm), which is approximately 9 times higher than the value measured in solution (Fig. 7). This improvement of CPL chirality in the KBr-dispersed pellet is considered to be due to the emergence of supramolecular chirality in the aggregated state.

**Fig. 8:**
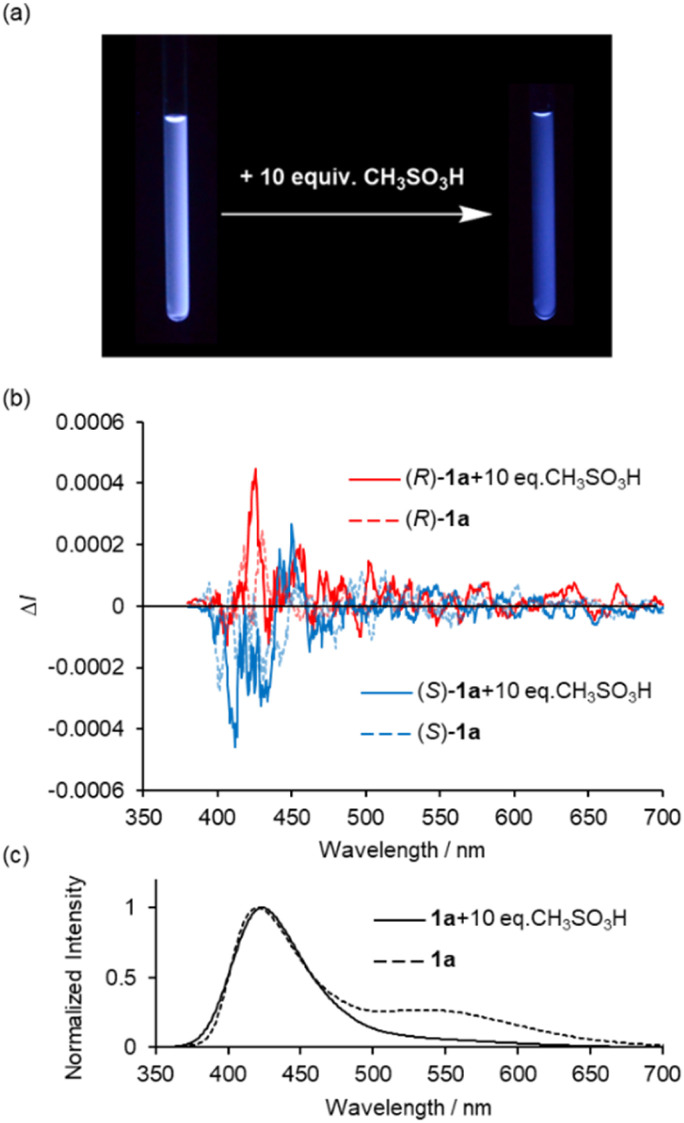
(a) Photographs under UV illumination at 365 nm, (b) CPL and (c) normalized total emission spectra of (*R*)- and (*S*)-1a in CH_2_Cl_2_ (2.0 × 10^−4^ M) with and without CH_3_SO_3_H at 298 K (*λ*_ex_ = 350 nm).

One of the most important photophysical properties of complex 1a is the acid-induced CPL enhancement, observed in CH_2_Cl_2_ upon addition of an excess CH_3_SO_3_H. As shown in Fig. 8a, the addition of excess amounts of CH_3_SO_3_H (10 equiv.) to a bluish-white emissive solution of complex 1a in CH_2_Cl_2_ typically causes the solution to exhibit blue fluorescence at 298 K. ^1^H NMR spectrum of complex 1a with CH_3_SO_3_H in CDCl_3_ suggests that 1a is stable under low concentrated acidic solution (Fig. S10†). Fig. 8b and c show changes in the CPL and total emission spectra of 2.0 × 10^−4^ M solutions of 1a in CH_2_Cl_2_. The addition of CH_3_SO_3_H resulted in an increase in CPL intensity around 420 nm (Fig. 8b). In the total emission spectrum, the emission band around 540 nm disappeared (Fig. 8c). The maximum emission |*g*_lum_| values in the CPL spectra were calculated to be 9.0 × 10^−4^ (426 nm), which is approximately 4 times higher than the value measured in the original solution. The UV-vis spectra of 1a in CH_2_Cl_2_ with varying equivalents of CH_3_SO_3_H are shown in Fig. S9 (ESI†), where the decrease of n–π* transition band around 380 nm was observed. Hence, the increase in CPL upon acid addition can be attributed to the decrease of n–π* character in the luminescence process.

### Theoretical calculations

**Fig. 9:**
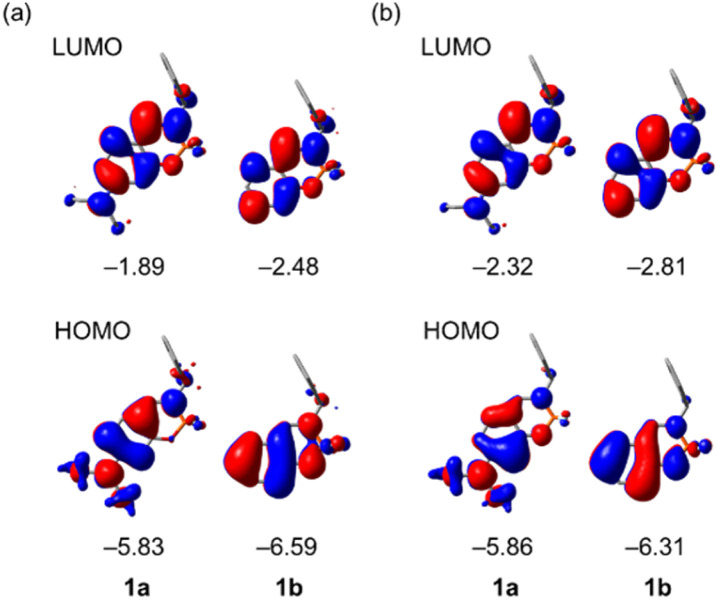
Molecular orbitals (overhead views) and eigenvalues [eV] for the frontier orbitals of (*S*)-1a and (*S*)-1b estimated from DFT calculations (B3LYP/6-31+G(d,p)) on the basis of the optimized geometries in the (a) S_0_ ground and (b) S_1_ excited states. Hydrogen atoms are omitted for clarity.

To get more insight into the photophysical properties of the present chiral boron complexes, we performed density functional theory (DFT) and time-dependent (TD) DFT calculations on the B3LYP/6-31+G(d,p) level, using the Gaussian 16 program. The optimized structures and frontier orbitals of (*S*)-1a and (*S*)-1b in the S_0_ (ground state) and S_1_ (excited state) states were estimated using DFT calculations on the basis of the X-ray structures (Fig. 9). The HOMOs are principally π orbitals of the ligand, including the non-bonding orbital of the diethylamino group of (*S*)-1a, whereas the LUMOs are in the ligand (π*). The energy levels and electronic configurations of the singlet states of these complexes were estimated from TD-DFT calculations (B3LYP/6-31+G(d,p)) (Tables S3 and S4, ESI†). The major contribution of the electronic configuration of the S_1_ states is the HOMO-to-LUMO transition, which implies that the present fluorescence is principally attributable to a mixture of n–π* and π–π* transitions of (*S*)-1a and the π–π* transition of (*S*)-1b. The S_1_-to-S_0_ transition energies for S_1_ states were calculated to be 2.98 eV (416 nm) for (*S*)-1a and 2.96 eV (419 nm) for (*S*)-1b, which is consistent with the emission peak maxima of the first emission band of the experimental spectra (*λ*_max_ = 420 nm for (*S*)-1a and *λ*_max_ = 433 nm for (*S*)-1b).

**Fig. 10:**
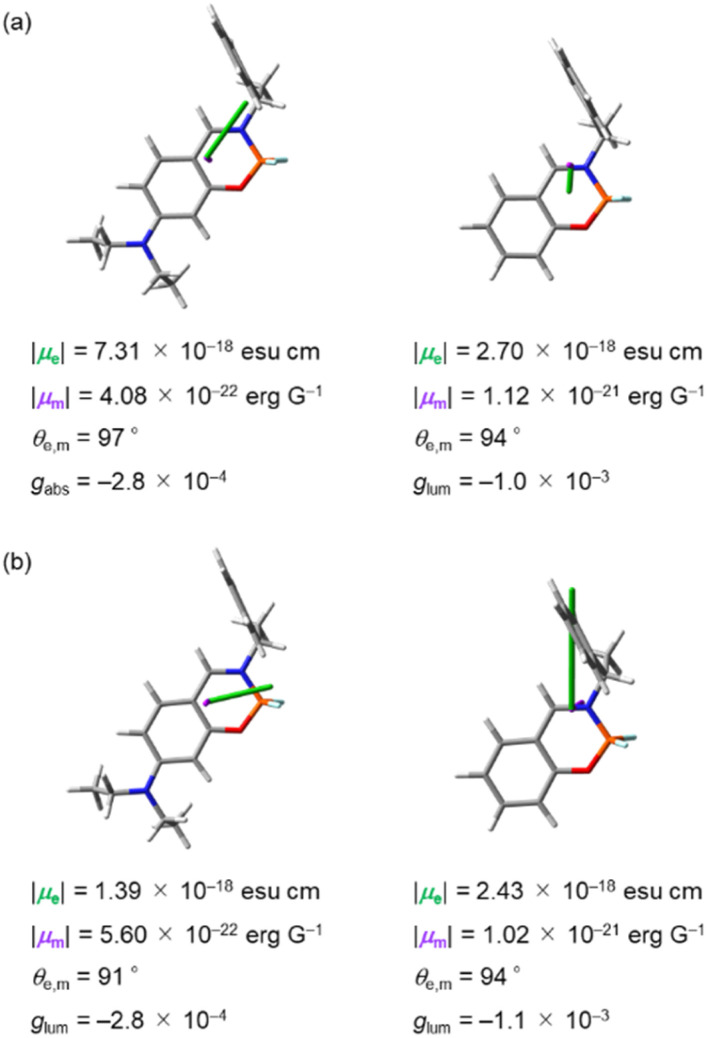
Electric (***μ***_e_, orange) and magnetic (***μ***_m_, purple) dipole moments of the (a) S_0_ → S_1_ transition and (b) S_1_ → S_0_ transition for (*S*)-1a (left) and (*S*)-1b (right) calculated at the B3LYP/6-31+G(d,p) level. Calculated values of transition dipole moments (|***μ***_e_|, |***μ***_m_| and *θ*_e,m_) and *g*_lum_ are given under each structure.

We considered the variation of chiroptical properties of (*S*)-1a and (*S*)-1b from the viewpoint of transition dipole moments using TD-DFT calculations. The dissymmetry factors *g*_abs_ for CD and *g*_lum_ for CPL are calculated with the following equation *g* = 4(|*μ*_e_||*μ*_m_|cos *θ*_e,m_)/(|*μ*_e_|^2^ + |*μ*_m_|^2^), where |*μ*_e_|, |*μ*_m_| and *θ*_e,m_ are the electric transition dipole moments, magnetic transition dipole moments and the angles between the two vectors *μ*_e_ and *μ*_m_, respectively.^28^ In the case of CPL-SOMs, |*μ*_m_| is basically much smaller compared with |*μ*_e_| and can be neglected. Thus, the equation of *g* can be replaced as follows: *g* = 4(|*μ*_m_|cos *θ*_e,m_)/|*μ*_e_|, in which the *g* value is directly proportional to |*μ*_m_| and inversely proportional to |*μ*_e_|. Fig. 10 shows the electric and magnetic dipole moments calculated for the upward S_0_-to-S_1_ and the downward S_1_-to-S_0_ transitions of (*S*)-1a and (*S*)-1b in the optimized geometries. For the upward S_0_-to-S_1_ transition, *g*_abs_ values were calculated to be −2.8 × 10^−4^ for (*S*)-1a and −1.0 × 10^−3^ for (*S*)-1b (Fig. 10a), which is consistent with the results from experimental CD spectra (−1.4 × 10^−4^ for (*S*)-1a and −1.0 × 10^−3^ for (*S*)-1b, Table 1). The scalar values |***μ***_e_| of (*S*)-1a is more than 2 times higher than that of (*S*)-1b, whereas |***μ***_m_| was less than half compared to that of (*S*)-1b. Following the equation of the *g* value, the decrease of the *g*_abs_ value for (*S*)-1a compared to that of (*S*)-1b is attributed to the changes in the scalar values |***μ***_e_| and |***μ***_m_|. For the downward S_1_-to-S_0_ transition, *g*_lum_ values were calculated to be −2.8 × 10^−4^ for (*S*)-1a and −1.1 × 10^−3^ for (*S*)-1b (Fig. 10b) which is consistent with the result from experimental CPL spectra (−2.8 × 10^−4^ for (*S*)-1a and −1.0 × 10^−3^ for (*S*)-1b, Table 1). The small *g*_lum_ for (*S*)-1a can be traced back to the nearly orthogonal electric and magnetic dipole moments: *θ*_e,m_ = 91°. The angle *θ*_e,m_ for the downward transition of (*S*)-1b is *θ*_e,m_ = 94°. This apparently small change in *θ*_e,m_ alone would influence the *g*_lum_ by a factor of 4 (as cos 94°/cos 91° = 0.07/0.0017). Given the calculation results, we can be certain that the orientation of dipole moments in the downward S_1_-to-S_0_ transitions is a key for the decrease in *g*_lum_ for (*S*)-1a compare to that of (*S*)-1b. The enhancement of the CPL intensity with the addition of CH_3_SO_3_H was also attributed to the changes in orientation of dipole moments, as the protonation of the diethylamino group reduces the contribution of the n–π* transition character.

## Conclusions

In summary, we have demonstrated external environment sensitive circularly polarized luminescence based on Schiff-base difluoride boron complexes. These chiral compounds exhibited solvatofluorochromism and acid-induced CPL enhancement in the solution state. The CPL intensity was also enhanced in the KBr-dispersed pellet state with a *g*_lum_ value of 2.4 × 10^−3^. DFT and TD-DFT calculations of the structures and electronic configurations of (*S*)-1a and (*S*)-1b revealed a relationship between molecular structure and photophysical properties. Theoretical consideration of the effect of solvent polarity for solvatofluorochromic behavior observed in 1a is now in progress.

## Author contributions

The project was conceived by Masahiro Ikeshita, who also directed all experiment work, theoretical calculation and wrote the manuscript. Hongxi He performed experimental works except for CPL measurements. Maho Kitahara measured CPL spectra. Yoshitane Imai and Takashi Tsuno gave constructive guidance for this study.

## Conflicts of interest

There are no conflicts to declare.

## Supplementary Material

RA-012-D2RA07386B-s001

RA-012-D2RA07386B-s002
