# Supplementary material for: External environment sensitive circularly polarized luminescence properties of a chiral boron difluoride complex
Source: RSC Adv. 2022 Dec 6;12(53):34790–6. doi: 10.1039/d2ra07386b (PMC9724127; doi:10.1039/d2ra07386b)
Supplement: RA-012-D2RA07386B-s001 [file RA-012-D2RA07386B-s001.pdf]

Electronic Supplementary Material (ESI) for RSC Advances.
This journal is © The Royal Society of Chemistry 2022

Supporting Information

External environment sensitive circularly polarized luminescence properties of chiral boron difluoride complex

Masahiro Ikeshita,*[a] Hongxi He,[a] Maho Kitahara,[b] Yoshitane Imai*[b] and Takashi Tsuno*[a]

[a] Department of Applied Molecular Chemistry, College of Industrial Technology, Nihon University, Narashino, Chiba 275-8575, Japan

[b] Department of Applied Chemistry, Faculty of Science and Engineering, Kindai University 3-4-1 Kowakae, Higashi-Osaka, Osaka 577-8502, Japan

Table of Contents

1. **Instrumentation and Materials**
2. **Synthetic Procedures and Characterization**
3. **Single Crystal X-ray Diffraction Analysis**
4. **Photophysical Properties**
5. **Computational Details**
6. **Cartesian Coordinates (in Å)**
7. **References**

1. Instrumentation and Materials

Instruments

Melting points were measured by a ATM-01 melting temperature measurement device (AS ONE Corporation). IR spectra were acquired with a JASCO FT/IR4100ST spectrometer. High-resolution mass spectrometry was recorded on Bruker micrOTOF II spectrometer. ^{1}H and ^{13}C NMR spectra were recorded on Bruker Avance III 500 spectrometers, TMS as internal standard. Elemental analyses were performed on a YANAKO MT-5 at A-Rabbit-Science Japan Co., Ltd. UV–vis absorption spectra were obtained on a SHIMADZU UV-1900i spectrophotometer. CD spectra were recorded on a JASCO J-720 spectropolarimeter. Emission spectra were obtained on a JASCO FP-6500 spectrometer. CPL spectra were obtained at room temperature using a JASCO CPL-300 spectrofluoropolarimeter. Optical rotation was measured on a Jasco DIP-370 digital polarimeter.

Materials

4-(Diethylamino)salicylaldehyde (TCI), (*R*)- and (*S*)-1-phenylethylamine (Kanto Chemical), salicylaldehyde (Kanto Chemical), boron trifluoride - ethyl ether complex (TCI), triethylamine (Fujifilm Wako) and dry dichloroethane (Kanto Chemical) were obtained from commercial sources and were used without further purification. The solid-state samples were prepared according to the standard procedure for obtaining glassy KBr matrices.[S1]

2. Synthetic Procedures and Characterization

General procedure for ligands

The ligands were prepared by condensation of corresponding aldehyde with (*R*)/(*S*)-1-phenylethylamine in boiling ethanol. The spectral data of **2b** were corresponded with published papers.[S2] The physical properties and spectroscopic data of the ligands (*R*)/(*S*)-**2a** and **2b** are as follows.

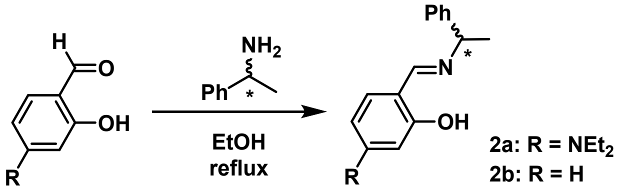

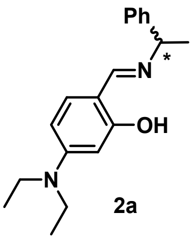

2a

(*R*)/(*S*)-2a: Brown liquid (98%); IR (KBr): $\nu = 3464$ (O-H), 1620 cm^{-1} (N=C); ^{1}H NMR (500 MHz, $CDCl_3$) $\delta = 13.96$ (br s, 1H), 8.09 (br s, 1H), 7.36–7.31 (m, 4H), 7.25–7.22 (m, 1H), 6.98 (d, $J = 8.5$ Hz, 1H), 6.15 (dd, $J = 8.5$, 2.3 Hz, 1H), 6.12 (d, $J = 2.3$ Hz, 1H), 4.48 (q, $J = 6.7$ Hz, 1H), 3.36 (q, $J = 7.0$ Hz, 4H), 1.59 (d, $J = 6.7$ Hz, 3H), 1.17 ppm (t, $J = 7.0$ Hz, 6H); ^{13}C NMR (125 MHz, $CDCl_3$) $\delta = 164.7$, 161.4, 150.8, 144.1, 132.4, 128.0, 126.4, 125.8, 107.9, 102.6, 97.6, 66.0, 43.9, 24.6, 12.1 ppm; Anal. calcd for $C_{19}H_{24}N_2O$: C, 76.99; H, 8.16; N, 9.45. Found: C, 76.98; H, 8.15; N, 9.43. (*R*)-**2a**: $[\alpha]_D^{25} = -247$ (c 0.001, $CHCl_3$), (*S*)-**2a**: $[\alpha]_D^{25} = +250$ (c 0.001, $CHCl_3$).

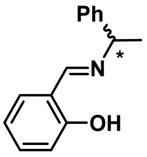

2b

(*R*)/(*S*)-2b[S2]**:** Yellow solid (98%); ^{1}H NMR (500 MHz, $CDCl_3$) $\delta = 13.53$ (s, 1H), 8.42 (s, 1H,), 7.39–7.25 (m, 7H,), 7.00 (d, $J = 8.2$ Hz, 1H), 6.89 (t, $J = 7.5$ Hz, 1H), 4.58 (q, $J = 6.7$ Hz, 1H), 1.65 (d, $J = 6.7$ Hz, 3H).

General procedure for boron complexes

A solution of corresponding chiral ligands (1 equiv.) in 1,2-dichloroethane was added triethylamine (4 equiv.) and boron trifluoride - ethyl ether complex (4 equiv.) The mixture was refluxed under nitrogen atmosphere for overnight. After that, the mixture was poured into water, and was extracted with dichloromethane, followed by drying over anhydrous $MgSO_4$. After the removal of the solvent, the crude product was purified by column chromatography (SiO_2) using dichloromethane as the eluent. After recrystallization from CH_2Cl_2/EtOH, (*R*)/(*S*)-**1a** and **1b** were obtained in moderate yields. The spectral data of **1b** were corresponded with published papers.[S2] The physical properties and spectroscopic data of the ligands (*R*)/(*S*)-**1a** and **1b** are as follows.

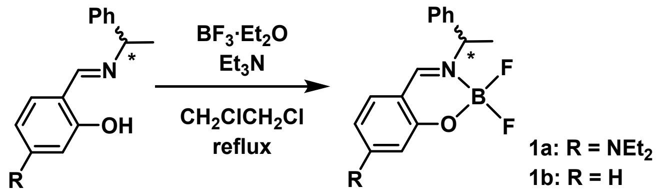

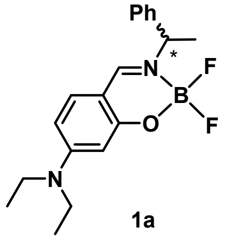

1a

(*R*)/(*S*)-1a: Colorless crystals (61%); m.p. 174–175 °C; IR (KBr): ν = 1628 cm^{-1} (N=C); ^1H NMR (500 MHz, $CDCl_3$) δ = 7.64 (br, 1H), 7.46–7.44 (m, 2H), 7.41–7.37 (m, 2H), 7.34–7.31 (m, 1H), 6.97 (d, *J* = 8.9 Hz, 1H), 6.23 (dd, *J* = 8.9, 2.4 Hz, 1H), 6.20 (d, *J* = 2.4 Hz, 1H), 5.29 (q, *J* = 7.0 Hz, 1H), 3.39 (q, *J* = 7.1 Hz, 4H), 1.79 (d, *J* = 7.0 Hz, 3H), 1.19 (t, *J* = 7.1 Hz, 6H) ppm; ^{13}C NMR (125 MHz, $CDCl_3$) δ = 160.6, 159.0, 155.3, 140.2, 133.0, 128.6, 127.9, 127.6, 105.8, 105.5, 97.8, 56.9, 44.8, 20.4, 12.4 ppm; HRMS (APCI+): m/z $[M]^+$ calcd for $C_{19}H_{23}N_2OBF_2$: 344.1869; found: 344.1840; Anal. calcd for $C_{19}H_{23}N_2OBF_2$: C, 66.30; H, 6.74; N, 8.14. Found: C, 66.02; H, 6.88; N, 8.18. (*R*)-**1a**: $[\alpha]_D^{25}$ = +222 (c 0.001, $CHCl_3$), (*S*)-**1a**: $[\alpha]_D^{25}$ = –226 (c 0.001, $CHCl_3$).

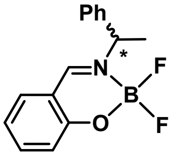

1b

(*R*)/(*S*)-1b[S2]: Colorless crystals (77%); ^1H NMR (500 MHz, $CDCl_3$) δ = 8.0 (br, 1H), 7.56 (ddd, *J* = 8.5, 7.2, 1.7 Hz, 1H), 7.48–7.38 (m, 5H), 7.24 (dd, *J* = 7.8, 1.7, 1H), 7.10 (d, *J* = 8.5 Hz, 1H), 6.90 (ddd, *J* = 7.8, 7.2, 0.9 Hz, 1H), 5.48 (q, *J* = 7.0 Hz, 1H), 1.87 (d, *J* = 7.0 Hz, 3H) ppm.

(a)

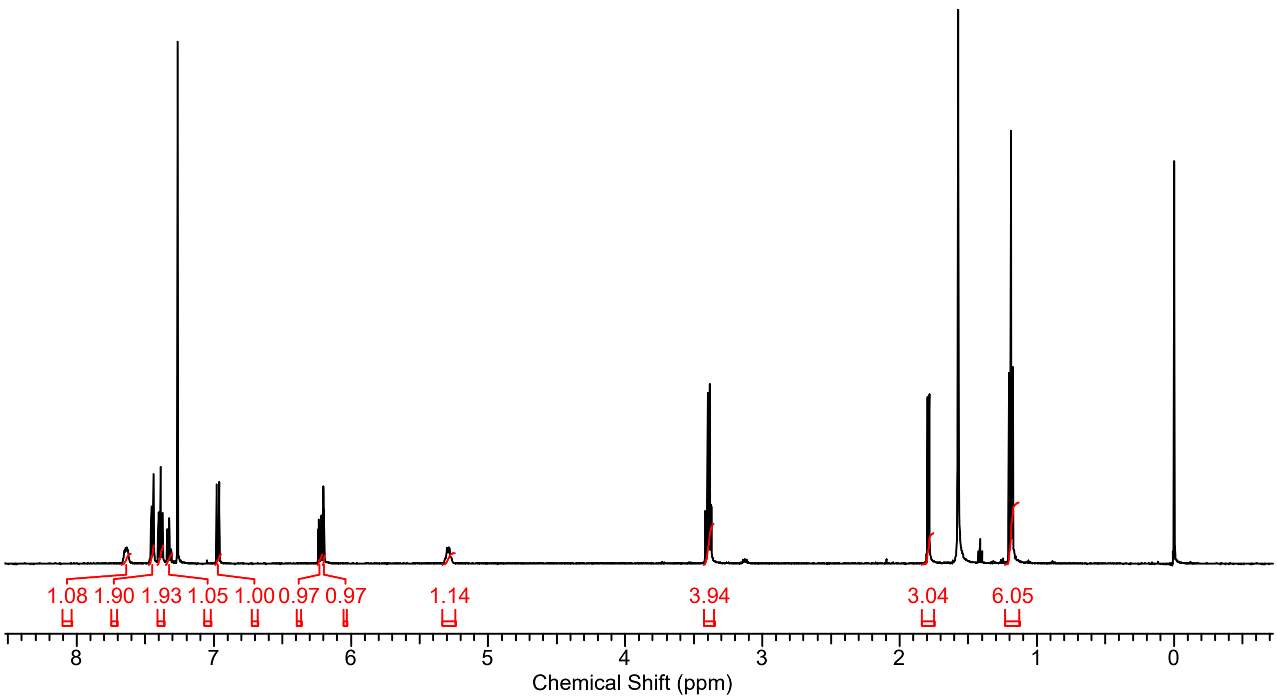

(b)

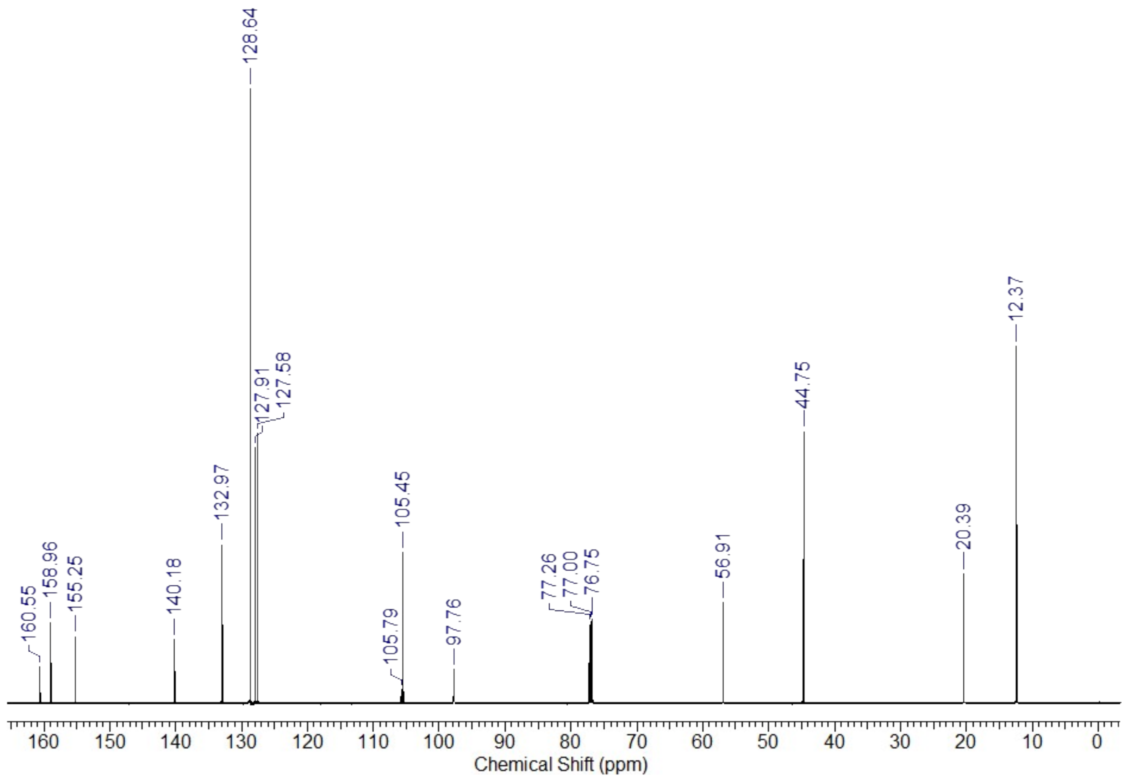

Fig. S1 (a) ^{1}H and (b) ^{13}C NMR spectra of (*S*)-**1a** in $CDCl_3$ (500 MHz, 298 K).

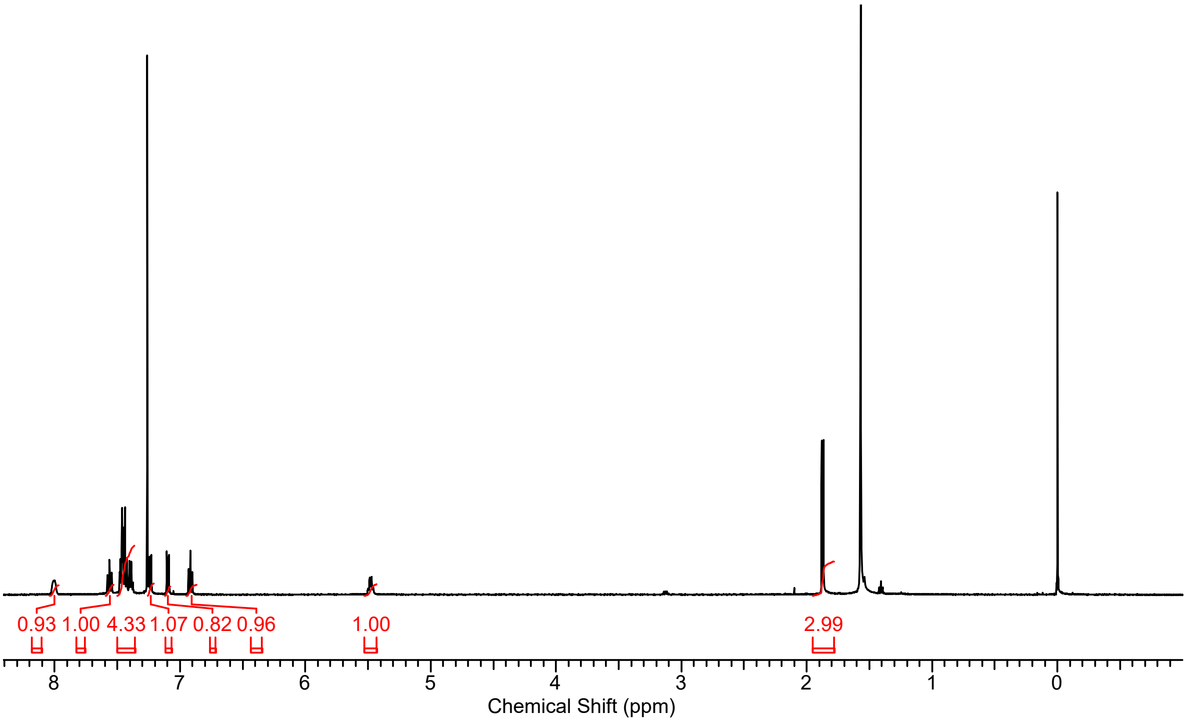

Fig. S2 ^{1}H NMR spectrum of (*S*)-**1b** in $CDCl_3$ (500 MHz, 298 K).

(a)

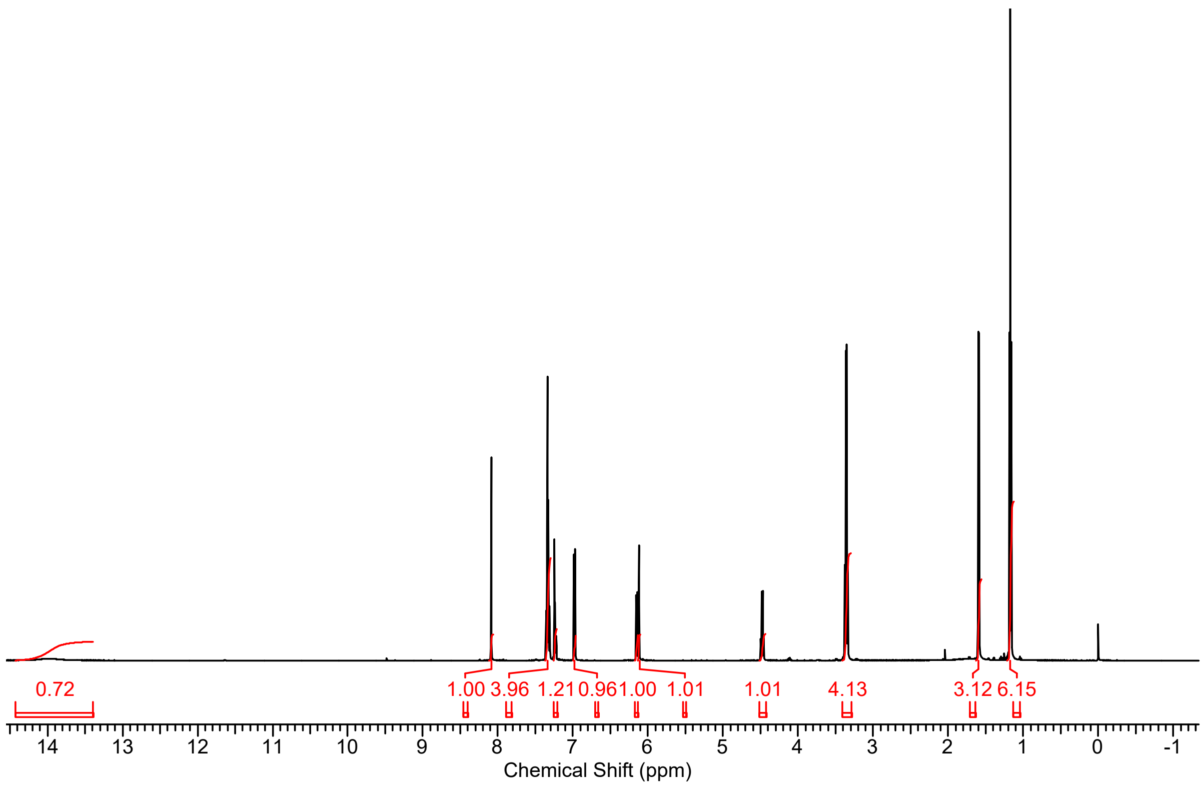

(b)

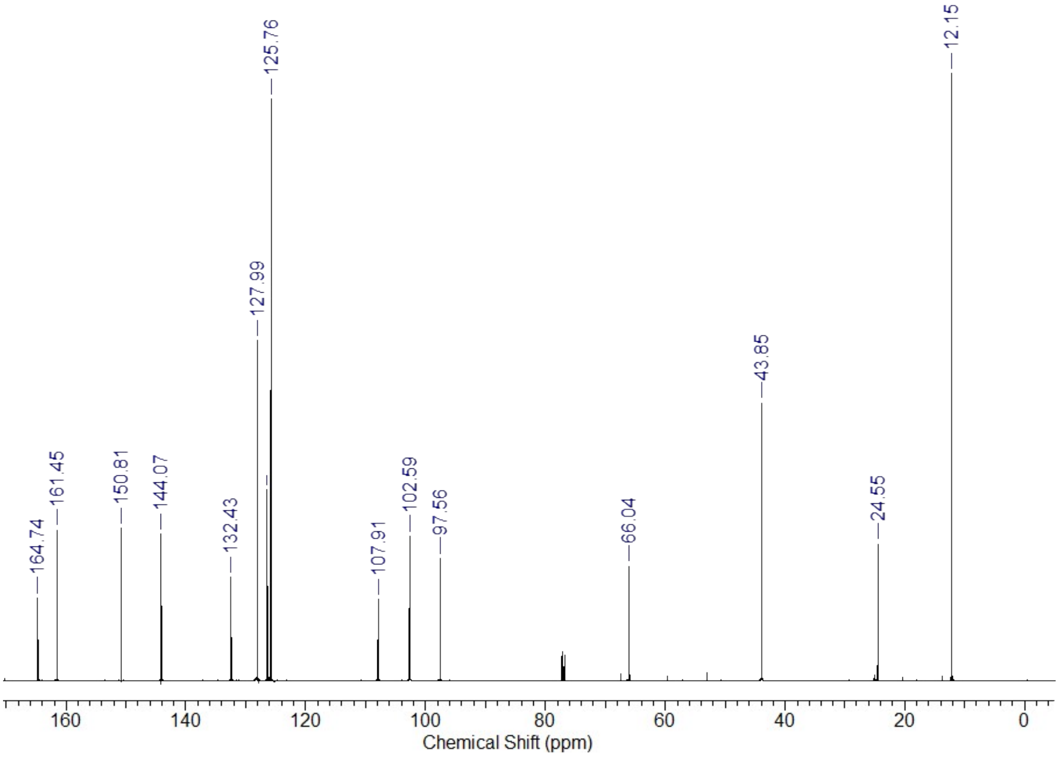

Fig. S3 (a) ^{1}H and (b) ^{13}C NMR spectra of (*S*)-**2a** in $CDCl_3$ (500 MHz, 298 K).

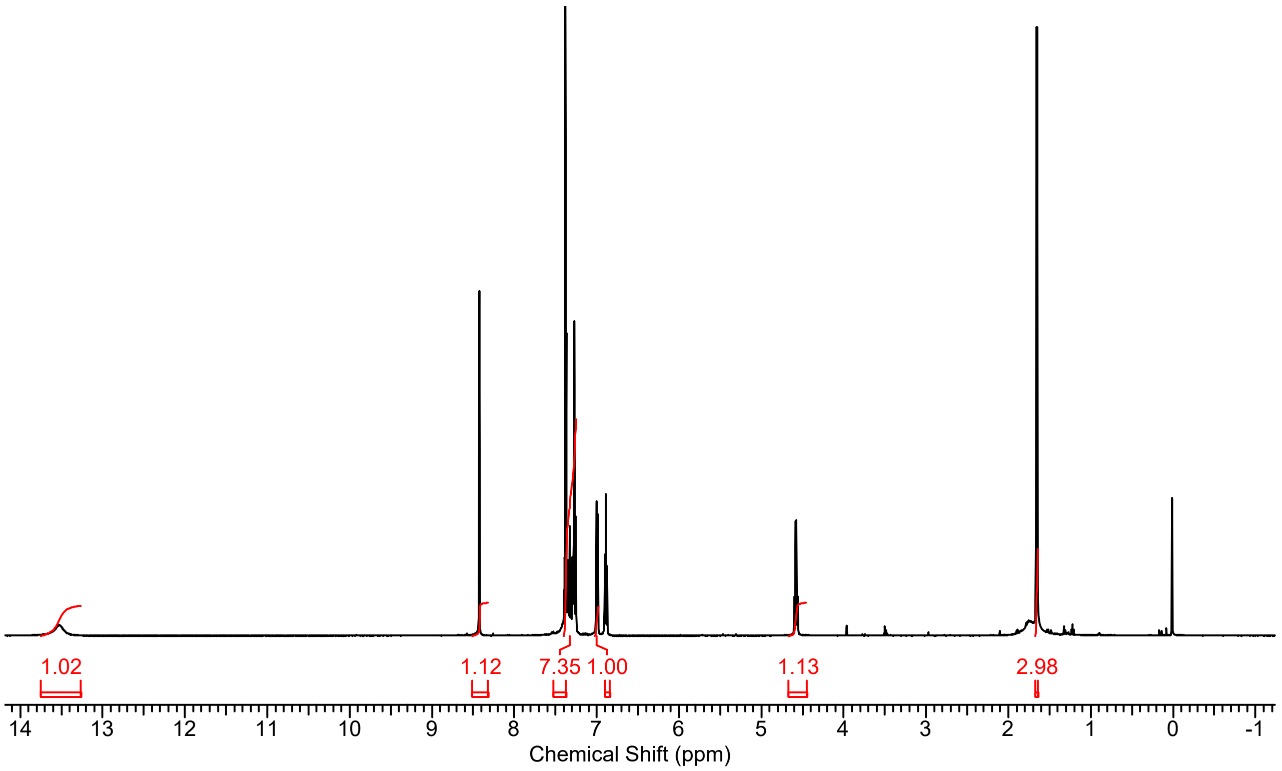

Fig. S4 ^{1}H NMR spectrum of (*S*)-**2b** in $CDCl_3$ (500 MHz, 298 K).

3. Single Crystal X-ray Diffraction Analysis

Crystals suitable for XRD studies were analyzed using Rigaku XtaLAB mini2 benchtop X-ray crystallography system equipped with a Mo rotating-anode X-ray generator with monochromated Mo-K_α radiation ($\lambda = 0.71073$ Å). The molecular structures and packings in crystals (*S*)-**1a** were solved by direct methods and refined using the full-matrix least-squares method. In subsequent refinements, the function $\Sigma\omega(F_o^2-F_c^2)^2$ was minimized, where F_o and F_c are the observed and calculated structure factor amplitudes, respectively. The positions of non-hydrogen atoms were found from difference Fourier electron density maps and refined anisotropically. All calculations were performed using the Crystal Structure crystallographic or CrysAlisPro program software package, and illustrations were drawn by using ORTEP.

(a)

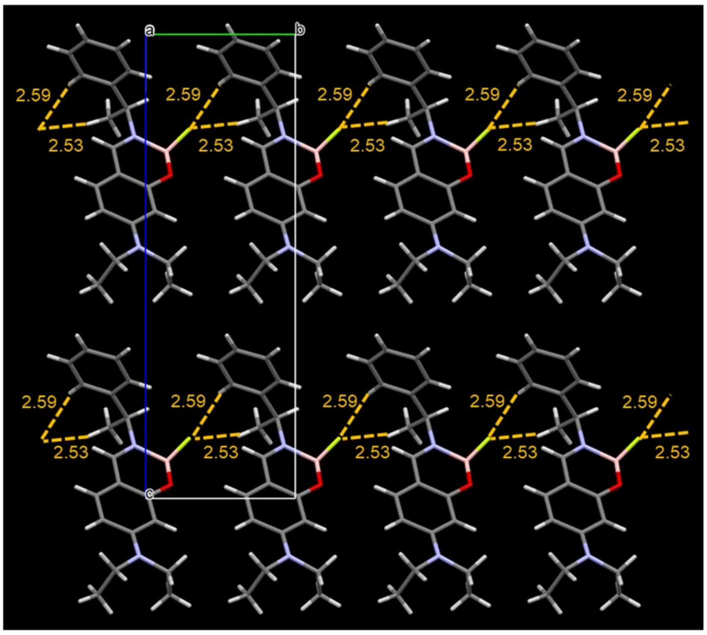

(b)

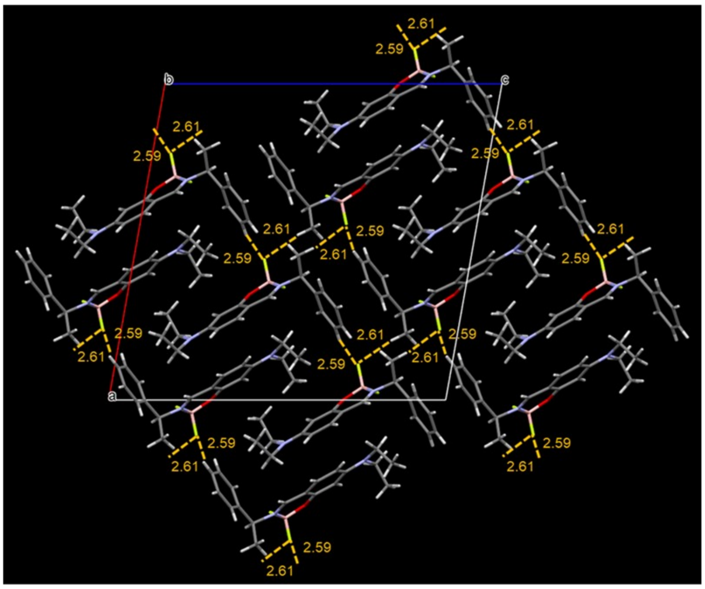

Fig. S5 Packing structure of (*S*)-**1a**. (a) The *a*-axis and (b) the *b*-axis projections showing H··F (orange broken lines) interactions.

Table S1. Crystal data and structural refinement details for complexes (*S*)-**1a**.

	(*S*)-**1a**
Formula	$C_{19}H_{23}BF_2NO$
M_F	344.20
T [K]	113.15
Crystal color, habit	colorless, plate
Crystal size [mm]	0.456×0.125×0.058
Crystal system	monoclinic
Space group	$P2_1$ (#4)
a [Å]	19.6617(11)
b [Å]	6.5542(4)
c [Å]	20.6217(11)
α [°]	90
β [°]	100.086(5)
γ [°]	90
V [Å^3]	2616.4(3)
Z	6
D_{calcd} [g cm^{-3}]	1.311
Abs coeff (mm^{-1})	0.095
Abs correct	multi-scan
Transmiss max/min	0.999/0.961
F (000)	1092
θ range (°)	4.012–61.002
Rflns/unique	53335/15069
R_{int}	0.0466
Data/params	15069/1/685
Largest diff. peak and hole (e Å^{-3})	0.24/–0.20
R_1 (I > 2σ(I))[a]	0.0461
wR_2 (all reflections)[b]	0.1213
Goodness of fit	1.002
CCDC No.	2205119

[a] $R_1 = \Sigma(|F_o|–|F_c|)/ \Sigma(|F_o|)$. [b] $wR_2 = [w(F_o^2–F_c^2)^2]/wF_o^2)^2]^{1/2}$.

4. Photophysical Properties

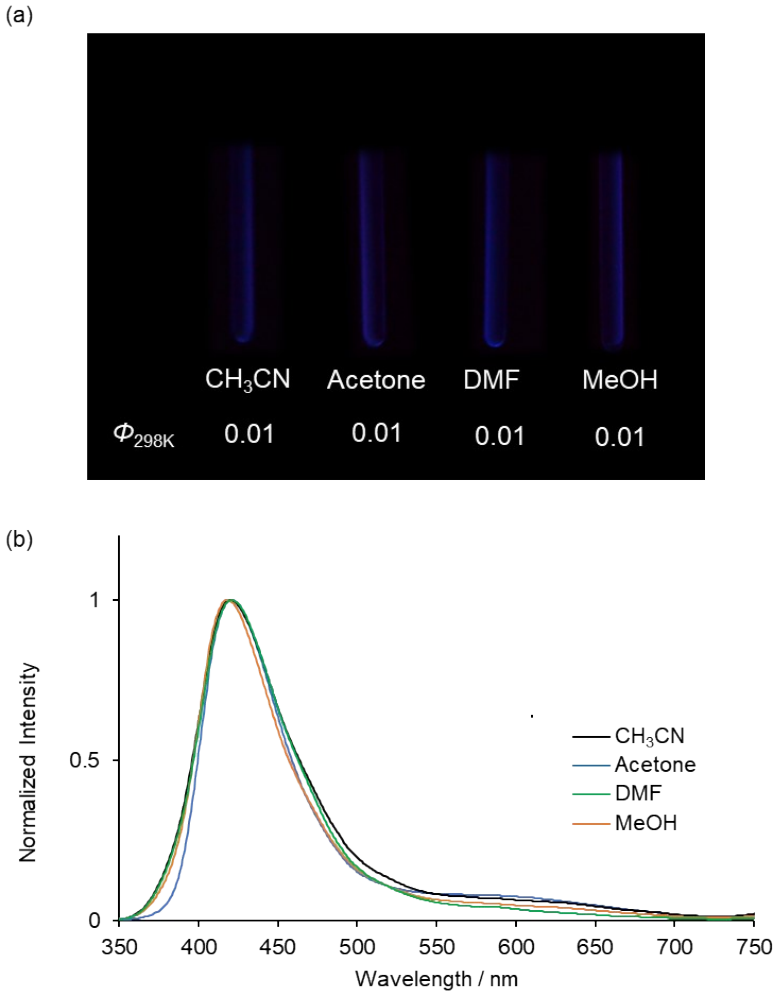

Fig. S6 (a) Photographs and (b) normalized emission spectra of 2.0 × 10^{-4} M solutions of (*S*)-**1a** in various polar organic solvents at 298 K.

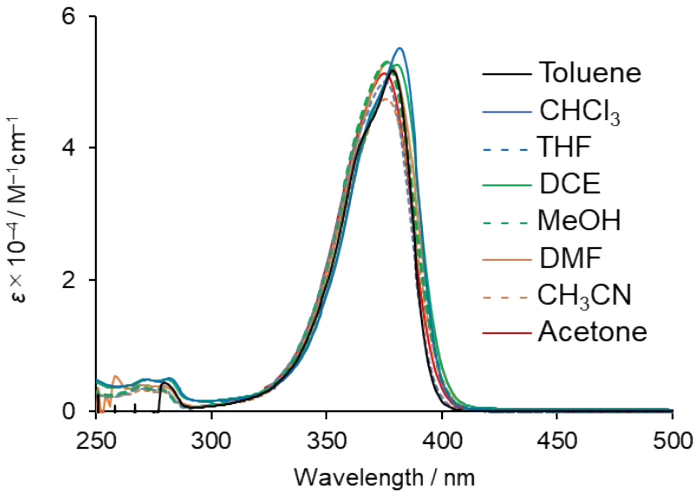

Fig. S7 UV-vis spectra of 2.0 × 10^{-4} M solutions of (*S*)-**1a** in various polar organic solvents at 298 K.

Table S2. Photophysical data for complexes (*S*)-**1a**.[a]

Solvents	λ_{abs} [nm]	λ_{max} [nm][b]	Φ[b,c]	CIE (x, y)[b]
CH_3CN	279, 376	420	0.01	0.19, 0.12
Acetone	375	421	0.01	0.20, 0.12
DMF	279, 377	421	0.01	0.17, 0.09
MeOH	278, 276	418	0.01	0.18, 0.10

[a] Data were obtained from a 2.0×10^{-4} M solution or crystals at 298 K. [b] λ_{ex} = 350 nm. [c] Luminescent quantum efficiencies measured using the absolute method with an integrating sphere.

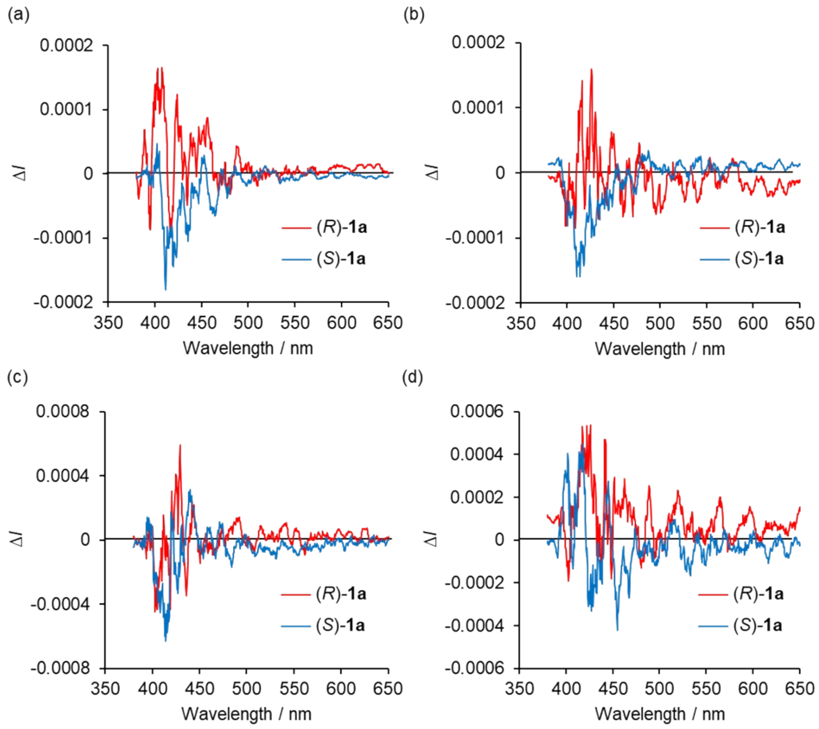

Fig. S8 CPL spectra of (*R*)-and (*S*)-**1a** in (a) toluene, (b) $CHCl_3$, (c) THF and (d) DCE (2.0 × 10^{-4} M) at 298 K (λ_{ex} = 350 nm).

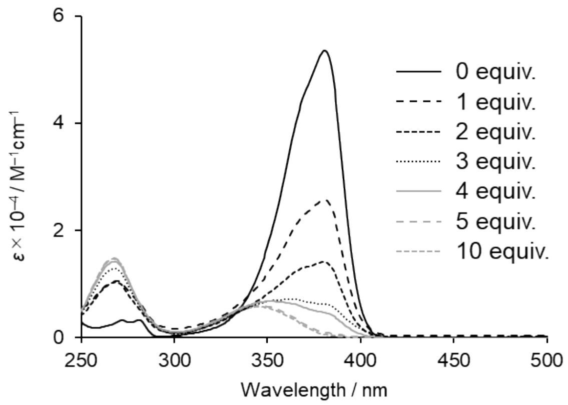

Fig. S9 Changes in UV-vis spectra of 2.0×10^{-4} M solutions of **1a** in CH_2Cl_2 as a function of equivalent of CH_3SO_3H at 298 K.

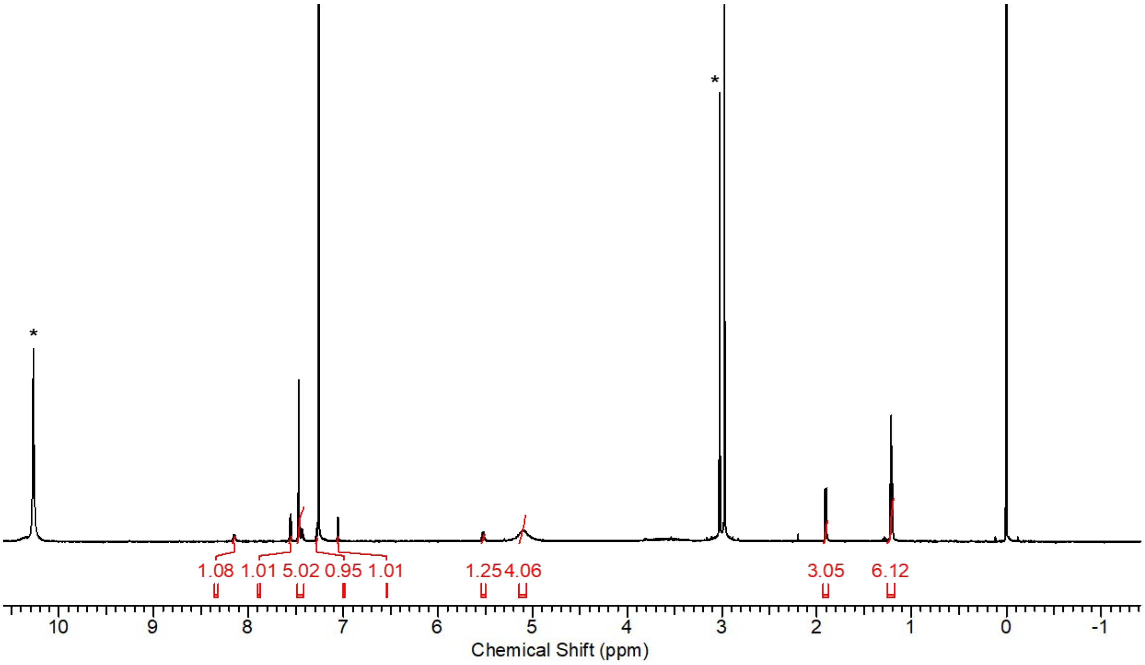

Fig. S10 ^1H NMR spectrum of (*S*)-**1a** with CH_3SO_3H (10 equiv.) in $CDCl_3$ (500 MHz, 2.0×10^{-3} M, 298 K). Asterisked peaks correspond to CH_3SO_3H.

5. Computational Details

All calculations were carried out based on DFT with the B3LYP exchange-correlation functional, using the Gaussian 16W program package.[S3] The basis set used was 6-31+G(d,p) for all atoms. Molecular orbitals and their eigenvalues for **1a** and **1b** were estimated using the optimized geometries determined by the DFT calculations using initial geometries obtained from XRD analysis. The singlet–singlet (*E*(Sn)) transition energies were estimated by time-dependent (TD) DFT calculation (B3LYP/6-31+G(d,p)).

Table S3. Selected data for excitation energy, major configuration, coefficient, and oscillator strength for **1** (for the geometries optimized in the S_0 state).[a]

Compound	State	Excitation energy (eV)	Major configuration[b]	Coefficient	Oscillator strength
(*S*)-**1a**	S_1	3.75 (330 nm)	HOMO→LUMO	0.695	0.7607
	S_2	3.80 (326 nm)	HOMO–1→LUMO	0.693	0.0223
	S_3	4.49 (276 nm)	HOMO–2→LUMO	0.700	0.0033
(*S*)-**1b**	S_1	3.58 (346 nm)	HOMO→LUMO	0.695	0.0987
	S_2	4.15 (299 nm)	HOMO–1→LUMO	0.687	0.0202
	S_3	4.33 (286 nm)	HOMO–2→LUMO	0.704	0.0006

[a] Estimated by TD-DFT (B3LYP/6-31+G(d,p)) calculations based on the optimized geometries. [b] Molecular orbitals are shown in Fig. 9.

Table S4. Selected data for excitation energy, major configuration, coefficient, and oscillator strength for **1** (for the geometries optimized in the S_1 state).[a]

Compound	State	Excitation energy (eV)	Major configuration[b]	Coefficient	Oscillator strength
(*S*)-**1a**	S_1	2.98 (416 nm)	HOMO→LUMO	0.528	0.0220
(*S*)-**1b**	S_1	2.96 (419 nm)	HOMO→LUMO	0.702	0.0666

[a] Estimated by TD-DFT (B3LYP/6-31+G(d,p)) calculations based on the optimized geometries. [b] Molecular orbitals are shown in Fig. 9.

6. Cartesian Coordinates (in Å)

Table S5. Cartesian coordinates (in angstrom) of (*S*)-**1a** in S_0 state[a].

atom	x	y	z
F	1.530002	2.2622	-1.538834
F	1.285901	3.212134	0.541792
O	-0.461483	1.910971	-0.321828
N	-4.507709	-0.620875	-0.503106
N	1.680075	0.831054	0.383342
C	5.357076	-2.361982	-1.029371
H	5.913958	-3.177435	-1.481863
C	4.712294	-1.416284	-1.835218
H	4.767994	-1.493717	-2.917311
C	4.001785	-0.367349	-1.253432
H	3.499434	0.364985	-1.880503
C	3.918566	-0.243226	0.14439
C	4.569186	-1.192497	0.941672
H	4.534579	-1.115269	2.023627
C	5.284299	-2.246241	0.358759
H	5.785362	-2.971226	0.994067
C	3.130205	0.930641	0.721695
H	3.464533	1.833595	0.204525
C	3.294243	1.170709	2.227308
H	2.726794	2.059111	2.512798
H	4.347343	1.335288	2.47543
H	2.927205	0.324028	2.817683
C	-3.162221	-0.535967	-0.223486
C	-2.434197	-1.68309	0.239624
H	-2.936401	-2.625379	0.407871
C	-1.083019	-1.600949	0.483673
H	-0.550041	-2.482322	0.832529
C	-0.365169	-0.397282	0.297578
C	-1.080813	0.751161	-0.152293
C	-2.453048	0.673614	-0.394173
H	-2.9368	1.584477	-0.717268
C	-5.253152	-1.869179	-0.31482
H	-4.647605	-2.706599	-0.67665
H	-6.124571	-1.824146	-0.974891
C	-5.258225	0.552662	-0.963539
H	-6.135498	0.18144	-1.501713
H	-4.655128	1.090261	-1.702673
C	-5.70746	1.510076	0.149625
H	-6.394715	1.016455	0.843341
H	-6.226588	2.370622	-0.286909
H	-4.855877	1.883793	0.724859
C	-5.717333	-2.134695	1.124655

H	-6.405802	-1.357046	1.468308
H	-4.872133	-2.166024	1.818411
H	-6.239239	-3.096958	1.177747
C	1.019208	-0.280416	0.569802
H	1.560521	-1.150371	0.94277
B	0.9923	2.107235	-0.254506

[a] The geometry was optimized by DFT calculation (B3LYP/6-31+G (d,p)).

Table S6. Cartesian coordinates (in angstrom) of (*S*)-**1b** in S_0 state[a].

atom	x	y	z
F	-0.452173	1.966054	-1.711147
F	-0.872283	2.99229	0.305301
O	-2.319354	1.302868	-0.428238
N	0.00646	0.737112	0.294408
C	-1.735159	-0.901751	0.300826
C	-2.671548	0.050064	-0.187643
C	-3.456976	-2.612538	0.343083
C	2.426842	0.138678	0.142931
C	-0.394049	-0.471951	0.546249
H	0.32107	-1.185033	0.957374
C	-2.145432	-2.229482	0.559821
H	-1.414436	-2.943451	0.931371
C	1.404769	1.17203	0.607494
H	1.54454	2.070339	0.00133
C	-4.005631	-0.349999	-0.395271
H	-4.713713	0.384627	-0.763028
C	3.210947	-0.610352	1.028814
H	3.117731	-0.465401	2.100298
C	-4.381545	-1.659989	-0.134495
H	-5.412688	-1.957265	-0.304076
C	2.590631	-0.062749	-1.238459
H	1.9876	0.515321	-1.934331
C	4.286977	-1.740465	-0.825304
H	5.006532	-2.463278	-1.19862
C	1.48843	1.58448	2.081931
H	1.295974	0.740427	2.752824
H	0.746158	2.359568	2.283276
H	2.481389	1.985953	2.305244
C	4.136193	-1.544794	0.547643
H	4.739004	-2.113906	1.249579
C	3.51007	-0.994262	-1.718865
H	3.625259	-1.134176	-2.789788
B	-0.941651	1.805676	-0.412965
H	-3.773572	-3.631456	0.538496

[a] The geometry was optimized by DFT calculation (B3LYP/6-31+G(d,p)).

Table S7. Cartesian coordinates (in angstrom) of (*S*)-**1a** in S_1 state[a].

atom	x	y	z
F	1.580725	2.284039	-1.507823
F	1.153551	3.143464	0.580888
O	-0.44401	1.773785	-0.448943
N	-4.575585	-0.594605	-0.554817
N	1.718119	0.795867	0.394698
C	5.476875	-2.256534	-1.102907
H	6.058527	-3.038713	-1.582617
C	4.759219	-1.335637	-1.875497
H	4.782849	-1.399403	-2.959959
C	4.015328	-0.331043	-1.25835
H	3.453428	0.379336	-1.859109
C	3.971647	-0.222381	0.142115
C	4.694722	-1.146685	0.904969
H	4.688123	-1.085084	1.988429
C	5.441741	-2.158424	0.287884
H	5.996607	-2.865104	0.899109
C	3.144634	0.911278	0.758499
H	3.484067	1.83846	0.286358
C	3.309405	1.089424	2.276537
H	2.73129	1.959527	2.596404
H	4.359649	1.254454	2.53823
H	2.945743	0.217133	2.829804
C	-3.235053	-0.586133	-0.205536
C	-2.555015	-1.662799	0.352945
H	-3.062224	-2.584031	0.604327
C	-1.155305	-1.579974	0.62538
H	-0.6874	-2.459087	1.062899
C	-0.362969	-0.462975	0.389313
C	-1.040036	0.647451	-0.171934
C	-2.441854	0.590485	-0.468313
H	-2.858576	1.481042	-0.91872
C	-5.351675	-1.822816	-0.398078
H	-4.716385	-2.66494	-0.695958
H	-6.174073	-1.784395	-1.119474
C	-5.286642	0.62427	-0.954286
H	-6.208848	0.300043	-1.444203
H	-4.710516	1.150554	-1.723501
C	-5.626478	1.584934	0.195306
H	-6.270926	1.105226	0.93746
H	-6.15221	2.46186	-0.198077
H	-4.724107	1.933578	0.706834
C	-5.916608	-2.062959	1.01212

H	-6.646152	-1.293739	1.281796
H	-5.126528	-2.060681	1.768126
H	-6.421635	-3.034486	1.047565
C	1.047847	-0.349549	0.680304
H	1.595658	-1.196817	1.073454
B	1.054163	2.023806	-0.241445

[a] The geometry was optimized by DFT calculation (B3LYP/6-31+G (d,p)).

Table S8. Cartesian coordinates (in angstrom) of (*S*)-**1b** in S_1 state[a].

atom	x	y	z
F	-0.339577	2.006923	-1.671629
F	-0.908054	2.944192	0.353769
O	-2.256254	1.262576	-0.553889
N	0.047533	0.726336	0.334354
C	-1.765078	-0.886353	0.371448
C	-2.655183	0.05375	-0.243197
C	-3.651626	-2.49338	0.372359
C	2.449285	0.110588	0.152112
C	-0.394762	-0.500528	0.66405
H	0.291363	-1.220052	1.094189
C	-2.281417	-2.131381	0.66159
H	-1.641798	-2.875683	1.1291
C	1.43047	1.127891	0.673023
H	1.597036	2.057113	0.120332
C	-4.00184	-0.29822	-0.528576
H	-4.623172	0.457668	-0.997489
C	3.278866	-0.641109	0.992458
H	3.22443	-0.516243	2.069137
C	-4.504511	-1.581488	-0.216549
H	-5.534433	-1.835228	-0.44011
C	2.559522	-0.070644	-1.237116
H	1.917403	0.506404	-1.897549
C	4.296602	-1.728325	-0.919795
H	5.010776	-2.43488	-1.332959
C	1.539113	1.460549	2.170107
H	1.325057	0.587899	2.795978
H	0.816332	2.241809	2.415906
H	2.542434	1.825555	2.41156
C	4.196831	-1.555631	0.460775
H	4.834074	-2.127404	1.129764
C	3.472856	-0.979944	-1.768834
H	3.546199	-1.102332	-2.845856
B	-0.836782	1.773519	-0.39032
H	-3.99058	-3.491875	0.627836

[a] The geometry was optimized by DFT calculation (B3LYP/6-31+G(d,p)).

7. References

[S1]. R. Kuroda and Y. Saito, Solid-State Circular Dichroism Spectra of Tris(diamine)cobalt(III) Complexes: Decomposition into E and A2 Components, *Bull. Chem. Soc. Jpn.*, 1976, **49**, 433–436.

[S2]. P. A. A. M. Vaz, J. Rocha, A. M. S. Silva and S. Guieu, Aggregation-Induced Emission Enhancement of Chiral Boranils, *New J. Chem.*, 2018, **42**, 18166–18171.

[S3]. Gaussian 16, Revision C.01, M. J. Frisch, G. W. Trucks, H. B. Schlegel, G. E. Scuseria, M. A. Robb, J. R. Cheeseman, G. Scalmani, V. Barone, G. A. Petersson, H. Nakatsuji, X. Li, M. Caricato, A. V. Marenich, J. Bloino, B. G. Janesko, R. Gomperts, B. Mennucci, H. P. Hratchian, J. V. Ortiz, A. F. Izmaylov, J. L. Sonnenberg, D. Williams-Young, F. Ding, F. Lipparini, F. Egidi, J. Goings, B. Peng, A. Petrone, T. Henderson, D. Ranasinghe, V. G. Zakrzewski, J. Gao, N. Rega, G. Zheng, W. Liang, M. Hada, M. Ehara, K. Toyota, R. Fukuda, J. Hasegawa, M. Ishida, T. Nakajima, Y. Honda, O. Kitao, H. Nakai, T. Vreven, K. Throssell, J. A. Montgomery, Jr., J. E. Peralta, F. Ogliaro, M. J. Bearpark, J. J. Heyd, E. N. Brothers, K. N. Kudin, V. N. Staroverov, T. A. Keith, R. Kobayashi, J. Normand, K. Raghavachari, A. P. Rendell, J. C. Burant, S. S. Iyengar, J. Tomasi, M. Cossi, J. M. Millam, M. Klene, C. Adamo, R. Cammi, J. W. Ochterski, R. L. Martin, K. Morokuma, O. Farkas, J. B. Foresman and D. J. Fox, Gaussian, Inc., Wallingford CT, 2016.
